# Sex-specific asymmetries in communication sound perception are not related to hand preference in an early primate

**DOI:** 10.1186/1741-7007-6-3

**Published:** 2008-01-16

**Authors:** Marina Scheumann, Elke Zimmermann

**Affiliations:** 1 Institute of Zoology, University of Veterinary Medicine Hannover, Bünteweg 17, 30559 Hannover, Germany; 2 Center for Systems Neuroscience, Bünteweg 17, 30559 Hannover, Germany

## Abstract

**Background:**

Left hemispheric dominance of language processing and handedness, previously thought to be unique to humans, is currently under debate. To gain an insight into the origin of lateralization in primates, we have studied gray mouse lemurs, suggested to represent the most ancestral primate condition. We explored potential functional asymmetries on the behavioral level by applying a combined handedness and auditory perception task. For testing handedness, we used a forced food-grasping task. For testing auditory perception, we adapted the head turn paradigm, originally established for exploring hemispheric specializations in conspecific sound processing in Old World monkeys, and exposed 38 subjects to control sounds and conspecific communication sounds of positive and negative emotional valence.

**Results:**

The tested mouse lemur population did not show an asymmetry in hand preference or in orientation towards conspecific communication sounds. However, males, but not females, exhibited a significant right ear-left hemisphere bias when exposed to conspecific communication sounds of negative emotional valence. Orientation asymmetries were not related to hand preference.

**Conclusion:**

Our results provide the first evidence for sex-specific asymmetries for conspecific communication sound perception in non-human primates. Furthermore, they suggest that hemispheric dominance for communication sound processing evolved before handedness and independently from each other.

## Background

A central question in evolutionary neuroscience is how and for what purpose did brain functions became lateralized. Left hemispheric dominance of language processing and handedness were previously thought to be unique for humans [1-5]. Therefore, it is suggested that language processing and handedness co-evolved and were linked to each other [5]. Men and women differ in the degree of lateralization, as well as in their linguistic and emotional skills [2,6,7].

Studies in non-human mammals using different techniques have questioned the view of human uniqueness (see for reviews [8,9]). Hemispheric comparison of the sizes of brain structures, relevant for language processing, revealed comparable anatomical asymmetries between humans and great apes in the *Planum temporale*, the Sylvian fissure and the Broca or homolog areas [10-17]. The latter is also involved in motor actions [4]. Sylvian fissure asymmetries similar to humans were also documented for Old World and some New World monkeys [15,18,19]. These findings suggest that these anatomical hemispheric asymmetries were already present on a pre-linguistic level.

Functional specializations of the two hemispheres reflected by handedness were found in non-human animals and humans. Various animal species exhibit individual hand/paw/foot preferences in tasks of different complexity (see, e.g. [20-26]). Thus, the degree of manual specialization was distinguished between individual hand/paw preference, meaning that a single individual used one hand/paw significantly more often than the other, and handedness, meaning that individuals of the whole population showed a significant bias in one direction [26]. Humans, some primates, some rodents, and even some toads showed right handedness/pawedness at population level in varying tasks (e.g. toads [20], rodents [27,28], non-human primates [29-31]) suggesting that asymmetries in motor control are shared between humans and non-human animals. In humans, right-handers showed a left hemispheric dominance for language. In chimpanzees, right-handedness in gestural communication is also present and enhanced when accompanied by vocalizations [32]. However, using imaging techniques, both right- and left-handed chimpanzees showed a left hemispheric asymmetry in the dimension of the *Planum temporale*, suggesting that human handedness and left hemispheric specialization for language evolved independently [33].

A left-hemispheric advantage for the perception of species-specific communication sounds similar to humans was described in birds and non-human mammals, based on behavioral and/or neurological approaches (humans [1], raptors [34], starlings [35], sea lions [36], mice [37-39], Japanese macaques [40-43], rhesus monkeys [44-47]; see for exceptions: vervet monkeys [48], barbary macaques [49]). To explore these hemispheric specializations in human infants and animals at the behavioral level, the head turn paradigm was established [44,50]. In the head turn paradigm, a sound is played back to the subject at exactly the same angle to both ears. An unconditioned behavioral response to the sound, the head turn, and its direction is taken as an indicator for an ear and hemispheric advantage in sound perception. As the connection of one ear to the contralateral hemisphere is dominant over the ipsilateral connection [51,52], a right head turn is taken as a behavioral indicator for the dominance of the left hemisphere and vice versa. The head turn paradigm has provided consistent evidence for asymmetries of communication sound perception of harpy eagles [34], sea lions [36] and anthropoid primates (rhesus macaques [44-46], vervet monkeys [48], but see [49] for a negative result). Rhesus and vervet monkeys showed a strong ear preference to conspecific communication sounds of varying emotional valence suggesting that a species-specific ear-hemispheric advantage is universal across primates [48]. To date, however, non-human primate research of asymmetries in the perception of communication sounds focused solely on Old World monkeys, whereas information on basal primates (prosimians) is lacking. Therefore it is unknown whether and to what extent an ancestral primate brain is already lateralized for communication sound perception and how this is related to handedness.

Here, we have studied the prosimian *Microcebus murinus*, suggested to represent the most ancestral primate condition [53], to gain first insight into the evolutionary roots of lateralization in the early primate brain. An individual hand preference in a food-reaching task was suggested based on a low sample size [54]. The lissencephalic mouse lemur brain is one of the simplest brains among extant primates [55]. It shows an anatomical asymmetry for the Sylvian fissure end point comparable to humans and apes [11,55].

The gray mouse lemur (*Microcebus murinus*) is a small-bodied, arboreal, nocturnal primate species living in a dispersed multi-male/multi-female social system [56,57]. Mouse lemurs produce audible and ultrasonic communication sounds, exhibit a high auditory sensitivity to a broad frequency range [58], and have developed an elaborate vocal repertoire [59-64]. Across species, specific call types are used in comparable contexts of their social life: e.g., social cohesion (e.g., trill), attention and alarm (e.g., whistle) or agonistic situations (e.g., tsak). Communication sounds emitted in social cohesion contexts are here termed as sounds with positive emotional valence, those emitted in attention/alarm and agonistic situations were termed sounds with negative emotional valence.

**Figure 1:**
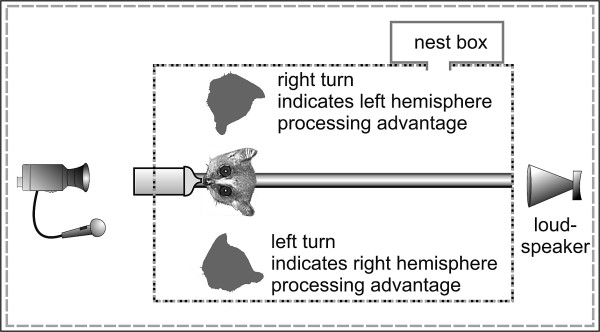
**Experimental setup**.

**Figure 2:**
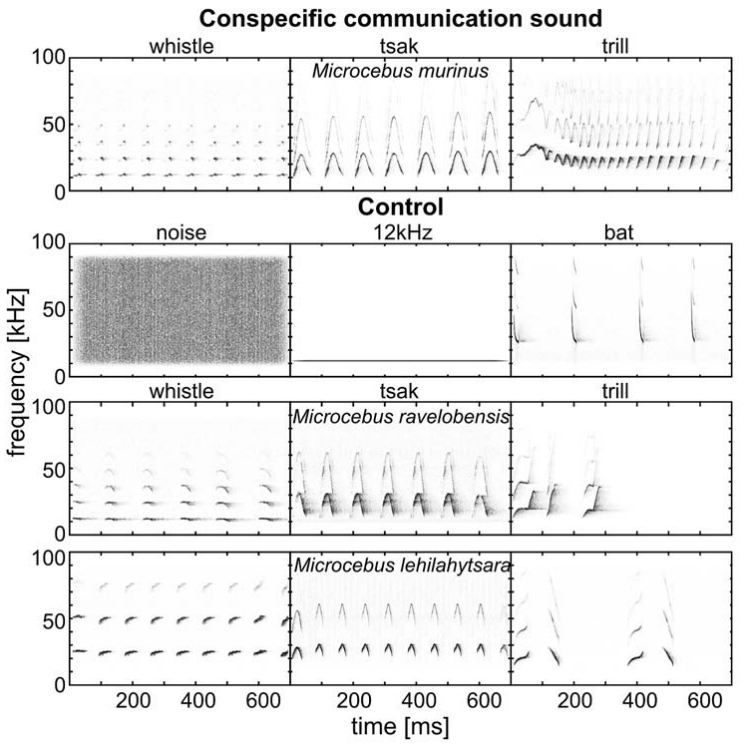
Spectrograms of the conspecific (*M. m. = Microcebus murinus) *and control playback stimuli (non-biological sound: noise, pure tone; heterospecific evolutionarily far related taxon: bat; heterospecific evolutionarily closely related taxon: *M. l*. = *M. lehilahytsara*; *M. r*. = *M. ravelobensis*).

By combing a forced food-grasping paradigm for handedness (= handedness test) with a head turn paradigm (= head turn test, Figure 1), we explored whether and to which extent these early primates showed asymmetries in hand usage and auditory perception and whether both are related. In the handedness test, subjects were forced to grasp meal worms with their hands out of a small hole in a box. In the head turn test, we played back acoustic stimuli from a loudspeaker 180° to the back of the subjects. We investigated subject's head turn direction in response to 12 acoustic stimuli (Figure 2): conspecific communication calls with positive (trill) and negative emotional valence (whistle and tsak) and controls. Controls were heterospecific communication calls of two evolutionarily closely related heterospecific *Microcebus *species (trill, whistle and tsak of *M. lehilahytsara *and *M. ravelobensis*), a heterospecific call of an evolutionarily far related species (bat) and non-biological sounds (noise, 12 kHz pure tone). Specifically, we hypothesized that: (1) gray mouse lemurs show handedness on individual level, but not at population level; (2) gray mouse lemurs show a hemispheric advantage for conspecific communication sounds, but not for controls, on the behavioral level as revealed for anthropoid primates; and (3) individual hand preference is not correlated with an potential orientation asymmetry.

## Results

### Hand preference

In the handedness test, 79% of the subjects (n = 30) showed an individual hand preference by using one hand significantly more often than the other (binominal test: p ≤ 0.049): 18 subjects were right-handed and 12 subjects were left-handed. However, at population level, we could not find any bias in the handedness index (HI) either for the whole population (mean_HI _= 0.125, SD = 0.752; one-sample t test: t = 1.027, df = 37, p = 0.311) or for one of the two sexes (females: mean_HI _= 0.174, SD = 0.755: t = 1.003, df = 18, p = 0.329; males: mean_HI _= 0.077, SD = 0.767, t = 0.437, df = 18, p = 0.668).

### Orientation asymmetry

**Figure 3:**
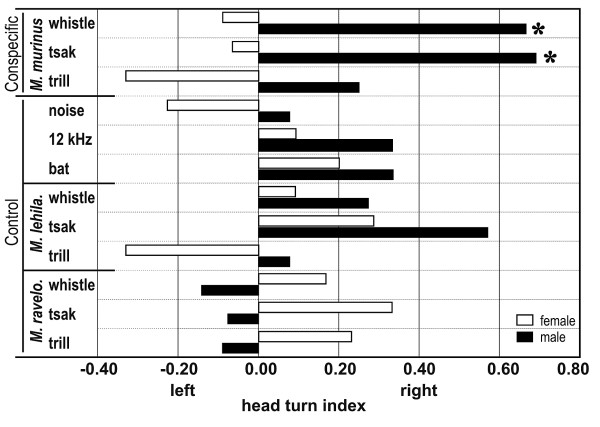
Head turn index of females and males for the conspecific (*M. m. = Microcebus murinus) *and control playback stimuli (non-biological sound: noise, pure tone; heterospecific evolutionarily far related taxon: bat; heterospecific evolutionarily closely related taxon: *M. l*. = *M. lehilahytsara*; *M. r*. = *M. ravelobensis*). * p < 0.05

Subjects turned their head in 86% of the conspecific communication calls and in 78% of the control trials. Analyzing the whole population, we did not find a head turn asymmetry for any conspecific or control playback stimuli (binominal test: p ≥ 0.185) except for the tsaks of the heterospecific *M. lehilahytsara *(binomial test: p = 0.036). The transgression probability for the control stimuli to obtain one significant result from nine single tests was p = 0.370, hence the significant results for the tsaks of *M. lehilahytsara *could be explained by chance. Interestingly, males and females differed in the orientation asymmetry toward conspecific communication sounds. Males showed a significantly right ear advantage for conspecific communication sounds of negative valence (binomial test: whistle, p = 0.039; tsak, p = 0.022; Table 1, Figure 3), but not for the conspecific communication sound of positive valence (binomial test: trill: p = 0.454). The transgression probability for the conspecific playback stimuli to obtain two significant results from three single tests was p = 0.007, therefore these orientation asymmetries cannot be explained by chance. Females did not show a significant head turn bias for any of the three conspecific call types (binomial test: p ≥ 0.388). Furthermore, neither males nor females showed a significant head turn preference for any of the control or heterospecific communication sounds (binomial test: males, p ≥ 0.057; females, p ≥ 0.424).

**Table 1:** Head turn index and number of subjects that did not turn their head (no) and that turned their head to the right side (R) or to the left side (L) for conspecific and control playback stimuli (non-biological sound; heterospecific evolutionarily far related species; heterospecific evolutionarily closely related species).

			**Males**			**Females**		
	**N**	**No**.	**R**	**L**	**Index**	**R**	**L**	**Index**

**Conspecific communication sounds**								

Whistle	31	8	10	2	0.67*	5	6	-0.09
Tsak	33	5	11	2	0.69*	7	8	-0.07
Trill	28	0	10	6	0.25	4	8	-0.33

**Non-biological sounds**								

Noise	37	11	7	6	0.08	5	8	-0.23
12 kHz	30	10	6	3	0.33	6	5	0.09

**Heterospecific evolutionarily far related species**								

Bat	31	9	8	4	0.33	6	4	0.20

**Heterospecific evolutionarily closely related species (*Microcebus lehilahytsara*)**								

Whistle	28	6	7	4	0.27	6	5	0.09
Tsak	32	4	11	3	0.57	9	5	0.29
Trill	29	7	7	6	0.08	3	6	-0.33

**Heterospecific evolutionarily closely related species (*Microcebus ravelobensis*)**								

Whistle	33	7	6	8	-0.14	7	5	0.17
Tsak	26	4	6	7	-0.08	6	3	0.33
Trill	27	3	5	6	-0.09	8	5	0.23

* p < 0.05

### Hand preference versus orientation asymmetry

Combining the results of the handedness and head turn test, we found no significant difference in the handedness index between subjects which turned their head to the right or left side for any of the playback stimuli (Mann-Whitney U = 50, p ≥ 0.079 for all stimuli). In addition, we restricted this analysis to the performance of males toward conspecific communication sounds of negative emotional valence. The results, however, did not differ (whistle: Mann-Whitney U = 3, p ≥ 0.133, n_1 _= 10, n_2 _= 2; tsak: Mann-Whitney U = 10, p ≥ 0.197, n_1 _= 11, n_2 _= 2).

## Discussion

Gray mouse lemurs showed individual hand preferences as well as sex-specific orientation asymmetries related to emotional valence of conspecific communication calls. Thus, males, but not females, exhibit a right ear-left hemispheric advantage toward conspecific communication calls of negative emotional valence. Hand preference of subjects was not related to orientation asymmetries.

Gray mouse lemurs, like humans and non-human animals (e.g. [8,65]), showed individual hand preferences. However, we did not find hand preferences at population level that coincided with results of Dodson et al [54] based on a lower sample size. Various studies of hand usage revealed task specific differences [25,30,66]. They hypothesized that low-level tasks such as food reaching in contrast to more complex high-level tasks such as bimanual manipulation are too simple to show handedness at population level. During insect capturing, mouse lemurs have to catch moving insects that are too large to handle with only one hand. Therefore they capture insects using their mouth and one or both hands (unpublished results), which suggest that they lack fine motor control. In our task, we forced subjects to use one hand instead of the mouth, which made our task difficult for them. We assume therefore that the forced food-grasping paradigm is not a task that is too simple for these animals. Altogether, our results suggest that brain asymmetries for the control of hand usage are already present in an early primate. Handedness at population level evolved later within the primate order. Further, the occurrence of handedness at population level in some rodents, birds and amphibians (e.g. [20,21,27,28]) suggests a convergent evolution in different taxonomic lineages.

The results for the hemispheric advantage in communication sound perception are consistent and cannot be explained by a small sample size, individual head turn preference or position of nest box and tail. In comparison with other captive settings studying lateralization of auditory perception (e.g. [36,48]) we used a large sample. We can rule out that subjects showed a general orientation asymmetry. Analysing the head turn direction of each subject across all stimuli revealed that only 6 of the 38 subjects showed a significant individual side preference over all stimuli (two male and four females; binomial test: p ≤ 0.039; all other subjects p ≥ 0.065). We can further exclude that subjects' head turns were influenced by the position of the tail, which is important for balance in arboreal species, or the nest box. We found no differences in head turn direction when the tail was bent to the right or left side of the subject, or the nest box was attached on the right or the left side of the cage.

In humans, non-human primates and non-primate animals, a left hemispheric advantage for perception of communication sounds was described (humans [1], raptor [34], starlings [35], sea lions [36], mice [37-39], Japanese macaques [40-43], rhesus monkeys [44-47]). Mouse lemurs showed a different pattern of hemispheric advantage as revealed for anthropoid primates (Japanese monkeys [40-42], rhesus monkeys [44-46], vervet monkey [48]). However, in anthropoid primates the direction of hemispheric asymmetries is plastic. Thus, Japanese monkeys and rhesus monkeys showed a left hemispheric advantage and vervet monkeys a right hemispheric advantage for communication sound perception. Altogether, results in non-human primates revealed three patterns of hemispheric asymmetries, a left hemispheric advantage for the perception of communication sounds of negative emotional valence in males of an early primate, a left hemispheric advantage in rhesus monkeys [44-46] and a right hemispheric advantage in vervet monkeys for communication sound processing of varying emotional valence [48]. Furthermore, non-primate animals demonstrated a right ear – left hemispheric advantage for conspecific communication sounds (raptors [34], starlings [35], mice [37-39], sea lions [36]). Altogether, it seems that lateralization of auditory perception at population level evolved gradually in primates. Furthermore, findings in sea lions, mice and birds favor the view of a convergent evolution of hemispheric asymmetries in primate and non-primate animals. This suggests a more complex evolutionary scenario of hemispheric specializations in language processing than previously assumed.

Focussing on males, we found that conspecific communication calls of negative valence cause a right head turn bias whereas the communication calls of positive valence did not cause any bias. This suggests that lateralisation in communication sound perception is linked to emotional valence as shown for humans (e.g. [67]). However, recent studies in humans and rhesus monkeys hypothesized that hemispheric advantages for communication sound processing are more affected by specific changes in spectral and temporal cues of the acoustic stream [1,68,69]. Thus, in humans, the left hemisphere seems to be specialized to the analyses of rapid temporal changes that speech perception requires whereas the right hemisphere is specialized to the analyses of fine-grained spectral changes that music perception requires [1,69]. According to the calls of the mouse lemurs whistles and tsaks are very short calls with a rapid repetition rate whereas the trills are long calls with a complex frequency structure. Further studies will explore to what extent these cues can explain orientation asymmetries in mouse lemurs.

In humans, men are more lateralized than women [2]. In mouse lemurs we found a right ear-left hemispheric advantage toward conspecific communication sounds of negative valence for males, but not for females or the whole population. This may suggest that as in humans, mouse lemur males are more lateralized than females. In humans, men and women differ in the performance of various linguistic and emotional tasks [7,70]. Thus, females perform better than males. Thereby, imaging studies revealed that during phonological processing, in men the left hemisphere is activated whereas in women both hemispheres are activated [71,72]. Further, sex differences occurred in the anatomy of language processing areas as well as in the interhemispheric connection, the corpus callosum [73]. The corpus callosum is larger in women than in men, suggesting more fibres that connect the two hemispheres. Kimura [7] suggested that either the functions of the two hemispheres are not sharply separated in women or that the larger commissural connections reduce the hemispheric differences. Such sex-specific anatomical differences in the corpus callosum were also found in prosimians, rats, dogs and apes, but not in New and Old World monkeys [74-77]. As in humans the corpus callosum is larger in females than in males of prosimians. Sex hormones are suggested to trigger these sex specific differences [2]. Based on this, we suggest that mouse lemur females did not exhibit an orientation bias because the strong commissural connections reduce lateralization by analysing communication sounds in both hemispheres.

It is suggested that human language evolved from manual and facial gestures rather than from animal vocal communication [4]. This is supported by the fact that right-handedness is related to left hemispheric dominance of speech processing [4]. However, in mouse lemurs we did not find a relation between individual hand preference and ear-hemispheric advantages toward conspecific communication sounds. Such a relationship is also lacking in non-human primates, who do show handedness at population level [29,44]. Thus, rhesus monkeys demonstrated a right-hand preference at population level in a coordinated bimanual task [29], but they did not show a relation between individual handedness and head turn direction [44]. Altogether, these data render support for the hypothesis that hand preference and lateralization of communication sound perception evolved independently from each other in primates [33].

## Conclusion

The results of our study demonstrated that early primates already showed hand preference on an individual level and sex-specific orientation asymmetries linked to emotional valence. We suggest that this represents a first step of hemispheric specialization in an evolutionary scenario for handedness and laterality of communication sound processing in primates. As a second step, some anthropoid primates showed handedness at the population level or hemispheric dominance for processing of communication calls of varying emotional valence, but independently from each other. The lateralization of these traits reached highest specialization in humans where both are linked to each other.

## Methods

### Subjects

We tested 38 adult gray mouse lemurs (19 females, 19 males) in our breeding colony, housed in the animal facility of the Institute of Zoology, University of Veterinary Medicine, Hanover (for details in housing conditions see [78]). All subjects were born in captivity. Their age ranged from 1 to 9 years. The subjects were socially experienced with other gray mouse lemurs and housed alone or in groups of two to three individuals in three different rooms. A total of 24 subjects shared the same room with another mouse lemur species, *Microcebus lehilahytsara*, at some stage in their life. Note *Microcebus lehilahytsara *was termed previously *Microcebus rufus*.

### Experimental setup

Each mouse lemur was tested alone in a test cage (Ebecco stainless steel cage for marmosets, 80 × 87 × 50 cm) in a sound-attenuated chamber. The cage was equipped with two wooden bars (for climbing and to position the subject in front of the bottle), a nest box and either a transparent box with a small opening for the handedness test (1 × 2 cm) or a bottle with banana/peach juice for the head turn test. A loudspeaker was placed 180° on the opposite side of the nipple of the juice bottle (Figure 1). To control for the effect of the nest box, it was placed either on the right (15 subjects) or the left (15 subjects) side of the cage or under the loudspeaker (8 subjects). The playback stimuli were played back using the NiDisk 1.33 software on a Toshiba laptop equipped with an D/A converter card (National Instruments, sampling frequency 500 kHz). The laptop was connected via an amplifier (Pioneer a-337) to a high frequency loudspeaker (Panasonic Leaf Tweeter EAS-Th400A, frequency range: 2–70 kHz). Subjects' behavior was videotaped using a digital camcorder (Sony DR-TRV 22E PAL, Nightshoot) linked to the tape output of a U-30 bat detector (Ultra Sound Advice) as external microphone. The camera was connected to a monitor outside the chamber where the experimenter sat and observed the subjects.

### Playback stimuli

We used 12 different acoustic stimuli of two categories (Figure 2) as playback stimuli: (1) conspecific communication calls: whistle, tsak and trill of *M. murinus*, (2) controls: heterospecific evolutionarily closely related species (whistle, tsak and trill of *M. lehilahytsara*; whistle, tsak and trill of *M. ravelobensis*), heterospecific evolutionarily far related species (bat) and non-biological sounds (random noise, 12 kHz pure tone). The random noise and the 12 kHz pure tone were generated using Signal 4.1. (Engineering Design, Berkeley, USA). The bat call was used from a demo version of Batsound 3.31 (Pettersson Electronics, Uppsala, Sweden). Calls of *M. lehilahytsara *were recorded in our animal facility whereas the calls of *M. ravelobensis *and *M. murinus *are field recordings supplied by Braune [79]. Two sets of the 12 stimuli were created and each set was presented to half of the subjects.

An experimental trial consisted of the presentation of a playback stimulus. All playback stimuli except for heterospecific trill calls consist of a sequence of three sounds separated by a constant interval. The duration of these sounds was standardized to the duration of the conspecific trill call as the longest continuous sound element, and the intersound interval to the mean intercall interval of the conspecific trill calls as the longest intercall interval (3600 ms). For the heterospecific trill calls we used the species-specific mean intercall interval between trills (*M. lehilahytsara*: 1100 ms; *M. ravelobensis*: 350 ms). All acoustic stimuli were diffused with a sound pressure level of 75 ± 1 dB at a distance of 1 m (RMS measurement, Brüel und Kjær Measuring Amplifier Type 2610).

### Procedure

We habituated each subject to the experimental setup and the experimental procedure before an experiment started. For the experiment, a subject was removed from its home cage, placed in a new nest box and attached to the test cage in a sound-attenuated chamber. Each subject performed two successive tests in one session: (1) Handedness test: a subject was forced to grasp 10 dead meal worms with their hands out of a small hole in a transparent box for 15 min, (2) Head turn test: the transparent box was exchanged by a bottle of juice. Subjects got the juice through licking on the nipple of the bottle. Subjects were either exposed to no sound (habituation for 45 min) or to playback stimuli (experiment). The handedness and the head turn test started as soon as the door to the sound attenuated chamber was closed. Each session was conducted at the beginning of the activity period of each subject.

#### Habituation criterion

We defined a subject as habituated when it grasped for meal worms within the first 5 min of the handedness test and licked on the nipple of the bottle within the first 5 min of the head turn test. When a subject reached the habituation criterion, we conducted the first experiment the next day.

#### Experiment

After a subject had performed the handedness test, it was exposed to the head turn test. An experimental trial of the head turn test consisted of the presentation of a playback stimulus. We started a playback stimulus when the subject was sitting in a defined position meaning that it was licking on the nipple of the bottle while keeping its head straight and its hands on the wooden bar. Thereby, the loudspeaker was 180° to the back of the subject. Within one session, four acoustic stimuli (= four experimental trials) were played back to the subject in a random order, one stimuli of the conspecific communication calls and three controls with a minimum inter-stimulus interval of 5 min. If the session could not be finished in more than 2 h, we tested the remaining acoustic stimuli from the session on a separate day. A subject needed a minimum of 3 days (= three sessions) to complete the experiment. Sessions were separated by a minimum of 2 days.

#### Data and video analysis

We digitized all video tapes using Pinnacle Studio 8 and analyzed them using Interact 3.1. (Mangold International GmbH). We conducted a frame-by-frame analysis (25 frames/s) for the handedness and the head turn tests separately. For the analysis of hand preference, we recorded the first hand the subject was using in a grasping bout. A grasping bout started with the first grasp of the subject and ended when it successfully retrieved a meal worm. A maximum of 10 grasping bouts could be analyzed per session. For the head turn test, we determined the exact time (Frame) the playback was started using Music Maker Deluxe 2005 Version 10.0 (Music Editor 2.01, Magix AG). This time point was transferred manually to Interact 3.1. We analyzed all experimental trials with regard to the head position at the start of the playback stimulus. As subjects sometimes did not turn their head in response to the first sound of a playback stimulus, but to the second or third, we determined the head position for these playback stimuli at the onset of the second or third sound within a trial. We selected all trials in which the head criterion (= the subject was licking on the nipple of the bottle while keeping its head straight and its hands on the wooden bar) was fulfilled for further analysis.

For the selected trials, we analyzed first head turn direction and tail position in the first 18 s after stimulus presentation. For each trial we scored the following head turn responses: no response, subjects did not turn head more than 90° to either of the two sides within 18 s of stimuli presentation; right turn, subject turned its head more than 90° to the right side; left turn, subject turned head more than 90° to the left side. Tail position was scored as right, more than 50% of the subjects tail was bent to the right side; left, more than 50% subjects tail was bent to the left side; straight, more than 50% of subjects tail is laying on the wooden bar.

To assess inter-observer reliability, a naïve person coded 20% of the trials (= 93 trials). The first author and the naïve person agreed in 99% of the trials for head turn direction, in 88% of the trials for tail position and in 96% of the trials for head position. We used the Kappa test to measure the agreement between two evaluations of two raters, the naïve person and the first author. A value of 1 indicates perfect agreement and a value of 0 indicates no better agreement than chance (SPSS 14). The results of the kappa test revealed that reliability was excellent for the head turn direction (kappa = 0.98) and the tail position (kappa = 0.82) and good for the head position (kappa = 0.69).

### Statistical analysis

We calculated the handedness index for each subject according to the formula HI = (number right – number left)/(number right + number left), with positive values reflecting right hand bias and negative values reflecting left hand bias [31]. Furthermore we tested whether subjects used one hand more often than expected by chance using the binominal test with 50% chance level. We defined animals as left- or right-hander or ambiguous: right-handers, subjects used significantly more often the right hand than expected by chance (positive handedness index); left-handers, subjects used significantly more often the left hand than expected by chance (negative handedness index); ambiguous, subjects did not use one hand significantly more often than expected by chance. Based on the individual handedness indices, we tested handedness at population level as well as for females and males separately using the one-sample t test.

We calculated the percentage of head turn responses for conspecific communication calls and controls across all subjects and the respective playback stimuli. For all trials, in which subjects showed a response toward the playback stimuli, we calculated the head turn index for each stimulus according to the formula HI = (number of subjects who turned their head right – number of subjects who turned their head left)/(number subjects who turned their head right + left). Positive values reflecting right head turn bias-left hemispheric advantage and negative values reflecting left head turn bias-right hemispheric advantage. We tested whether significantly more subjects turned their head to one side than expected by chance for each of the 12 acoustic stimuli using the binomial test with 50% chance level for the whole population as well as for males and females, separately. To control for multiple testing, we used a method by Bortz, Lienert and Boehnke [80]. Thus, using a cumulative binomial distribution function, we calculated the binomial transgression probability *p *to obtain at least *k *significant results out of n tests. The observed significant results could not be explained by chance if *p *was smaller than the accepted global α error of 0.05. Furthermore we tested whether playback stimulus set, position of tail and of nest box influenced the side of the head turn using the Fisher's exact test for each of the 12 acoustic stimuli. We did not find a significant difference of head turn directions between the two stimulus sets (Fisher's exact test: p ≥ 0.1671 for all stimuli), right or left placed tails (Fisher's exact test: p ≥ 0.061 for all stimuli) or right or left attached nest boxes (Fisher's exact test: p ≥ 0.179 for all stimuli), therefore we could rule out that these factors affect the direction of head turns toward the playback stimuli.

We tested whether the handedness index differs between subjects that turned their head to the right and to the left side, respectively, for each of the 12 acoustic stimuli using the Mann-Whitney U test. All statistical tests were calculated using SPSS 14.

## Authors' contributions

MS participated in the design of the study, conducted the experiments, performed the video and statistical analysis and prepared the manuscript. EZ initiated, financed, mentored the study and contributed to the design of the study and the preparation of the manuscript. All authors have read and approved this manuscript.

## References

[B1] Zatorre RJ, Belin P, Penhune VB (2002). Structure and function of auditory cortex: music and speech. Trends Cogn Sci.

[B2] Toga AW, Thompson PM (2003). Mapping brain asymmetry. Nat Rev Neurosci.

[B3] Friederici A, Alter K (2004). Lateralization of auditory language functions: a dynamic dual pathway model. Brain Lang.

[B4] Corballis MC (2003). From mouth to hand: gesture, speech, and the evolution of right- handedness. Behav Brain Sci.

[B5] Halpern ME, Güntürkün O, Hopkins WD, Rogers LJ (2005). Lateralization of the vertebrate brain: taking the side of model systems. J Neurosci.

[B6] Falk D (1987). Brain lateralization in primates and its evolution in hominids. Yearb Phys Anthropol.

[B7] Kimura Doreen Sex and Cognition.

[B8] Vallortigara G, Rogers LJ (2003). Survival with an asymmetrical brain: advantages and disadvantages of cerebral lateralization. Behav Brain Sci.

[B9] Vallortigara G (2006). Cerebral lateralization: a common theme in the organization of the vertebrate brain. Cortex.

[B10] Le May M, Geschwind N (1975). Hemispheric differences in the brain of great apes. Brain Behav Evol.

[B11] Le May M (1976). Morphological cerebral asymmetries of modern man, fossil man, and nonhuman primate. Ann NY Acad Sci.

[B12] Yeni-Komishan GH, Benson DA (1976). Anatomical study of cerebral asymmetry in the temporal lobe of humans, chimpanzees, and rhesus monkeys. Science.

[B13] Gannon PJ, Holloway RL, Broadfield DC, Braun AR (1998). Asymmetry of chimpanzee planum temporale: Humanlike pattern of Wernicke's brain language area homolog. Science.

[B14] Hopkins WD, Marino L, Rilling JK, MacGregor LA (1998). Planum temporale asymmetries in great apes as revealed by magnetic resonance imaging (MRI). Neuroreport.

[B15] Hopkins WD, Pilcher DL, MacGregor LM (2000). Sylvian fissure asymmetries in nonhuman primates revisited: a comparative MRI study. Brain Behav Evol.

[B16] Cantalupo C, Hopkins WD (2001). Asymmetric Broca's area in great apes: a region of the ape brain is uncannily similar to one linked with speech in humans. Nature.

[B17] Cantalupo C, Pilcher DL, Hopkins WD (2003). Are planum temporale and sylvian fissure asymmetries directly related? A MRI study in great apes. Neuropsychologia.

[B18] Falk D (1978). Cerebral asymmetry in Old World monkeys. Acta Anat.

[B19] Heilbroner PL, Holloway RL (1988). Anatomical brain asymmetries in New World and Old World monkeys: stages of temporal lobe development in primate evolution. Am J Phys Anthropol.

[B20] Bisazza A, Cantalupo C, Robins A, Rogers L, Vallortigara G (1996). Right-pawedness in toads. Nature.

[B21] Rogers LJ, Workman L (1993). Footedness in birds. Anim Behav.

[B22] Izawa EI, Kusayama T, Watanabe S (2005). Foot-use laterality in the Japanese jungle crow (*Corvus macrorhynchos*). Behav Process.

[B23] Vallortigara G, Rogers LJ, Bisazza A (1999). Possible evolutionary origins of cognitive brain lateralization. Brain Res Rev.

[B24] Biddle FG, Eales BA (1996). The degree of lateralization of paw usage (handedness) in the mouse is defined by three major phenotypes. Behav Genet.

[B25] Fagot J, Vauclair J (1991). Manual laterality in nonhuman primates: a distinction between handedness and manual specialization. Psychol Bull.

[B26] McGrew WC, Marchant LF (1997). On the other hand: current issues in and meta-analysis of the behavioural laterality of hand function in nonhuman primates. Yearb Phys Anthropol.

[B27] Tang AC, Verstynen T (2002). Early life enviroment modulates handedness in rats. Behav Brain Res.

[B28] Güven M, Elalmis DD, Binokay S, Tan Ü (2003). Population-level right-paw preference in rats assessed by a new computerized food-reaching test. Int J Neurosci.

[B29] Westergaard GC, Suomi SJ (1996). Hand preference for a bimanual task in tufted capuchins (*Cebus apella*) and rhesus macaque (*Macaca mulatta*). J Comp Psychol.

[B30] Vauclair J, Meguerditchian A, Hopkins WD (2005). Hand preference for unimanual and coordinated bimanual tasks in baboons (*Papio anubis*). Cognit Brain Res.

[B31] Lonsdorf EV, Hopkins WD (2005). Wild chimpanzees show population-level handedness for tool use. Proc Natl Acad Sci USA.

[B32] Hopkins WD, Cantero M (2003). From hand to mouth in the evolution of language: the influence of vocal behaviour on lateralized hand use in manual gestures by chimpanzees (*Pan troglodytes*). Dev Sci.

[B33] Hopkins WD, Cantalupo C (2004). Handedness in chimpanzees (*Pan troglodytes*) is associated with asymmetries of the primary motor cortex but not with homologous language areas. Behav Neurosci.

[B34] Palleroni A, Hauser M (2003). Experience-dependent plasticity for auditory processing in a raptor. Science.

[B35] George I, Cousillas H, Richard J-P, Hausberger M (2002). Song perception in the European starling: hemispheric specialisation and individual variations. C R Biol.

[B36] Böye M, Güntürkün O, Vauclair J (2005). Right ear advantage for conspecifics calls in adults and subadults but not infants, California sea lions (*Zalophus californianus*): hemispheric specialization for communication?. Eur J Neurosci.

[B37] Ehret G (1987). Left hemisphere advantage in the mouse brain for recognizing ultrasonic communication calls. Nature.

[B38] Haase H, Ehret G, Elsner N, Roth G (1990). Lateralization of sound perception in the brain of the mouse (*Mus musculus*). Brain – Perception-Cognition: Proceedings of the 18th Göttinger Neurobiology Conference.

[B39] Geissler DB, Ehret G (2004). Auditory perception vs. recognition: representation of complex communication sounds in the mouse auditory cortical fields. Eur J Neurosci.

[B40] Petersen MR, Beecher MD, Zoloth SR, Moody DB, Stebbins WC (1978). Neural lateralization of species-specific vocalizations by Japanese macaques (*Macaca fuscata*). Science.

[B41] Petersen MR, Beecher MD, Zoloth SR, Green S, Marler PR, Moody DB, Stebbins WC (1984). Neural lateralization of vocalizations by Japanese macaques: communicative significance is important than acoustic structure. Behav Neurosci.

[B42] Beecher MD, Petersen MR, Zoloth SR, Moody DB, Stebbins WC (1979). Perception of conspecific vocalizations by Japanese macaques: evidence for selective attention and neural lateralization. Brain Behav Evol.

[B43] Heffner HE, Heffner RS (1986). Effect of unilateral and bilateral auditory cortex lesions on the discrimination of vocalizations by Japanese macaques. J Neurophysiol.

[B44] Hauser MD, Anderson K (1994). Left hemisphere dominance for processing vocalizations in adult, but not infant rhesus monkeys: field experiments. Proc Natl Acad Sci USA.

[B45] Hauser MD, Agnetta B, Perez C (1998). Orienting asymmetries in rhesus monkeys: the effect of time-domain changes on acoustic perception. Anim Behav.

[B46] Ghazanfar AA, Smith-Rohrberg D, Hauser MD (2001). The role of temporal cues in rhesus monkey vocal recognition: orienting asymmetries to reversed calls. Brain Behav Evol.

[B47] Poremba A, Malloy M, Saunders RC, Carson RE, Herscovitch P, Mishkin M (2004). Species-specific calls evoke asymmetric activity in the monkey's temporal poles. Nature.

[B48] Gil-da-Costa R, Hauser MD (2006). Vervet monkeys and humans show brain asymmetries for processing conspecific vocalizations, but with opposite patterns of laterality. Proc Roy Soc B-Biol Sci.

[B49] Teufel C, Hammerschmidt K, Fischer J (2007). Lack of orienting asymmetries in barbary macaque: implications for studies of lateralized auditory processing. Anim Behav.

[B50] Ecklund-Flores L, Turkewitz G (1996). Asymmetric headturning to speech and nonspeech in human newborns. Dev Psychobiol.

[B51] Petersen MR, Snowdon CT, Brown CH, Petersen MR (1982). The perception of species-specific vocalizations by primates: a conceptual framework. Primate Communication.

[B52] Heffner HE, Heffner RS (1990). Effect of bilateral auditory cortex lesions on absolute thresholds in Japanese macaques. J Neurophysiol.

[B53] Martin RD, Charles-Dominique P, Martin RD (1972). A preliminary field-study of the lesser mouse lemur (*Microcebus murnius*, Miller 1977). Behavioral and Ecology of Nocturnal Prosimians.

[B54] Dodson DL, Stafford D, Forsythe C, Seltzer CP, Ward JP (1992). Laterality in quadrupedal and bipedal prosimians: reach and whole-body turn in the mouse lemur (*Microcebus murinus*) and the Galago (*Galago moholi*). Am J Primatol.

[B55] De Lacoste M-C, Horvath DS, Woodward DJ (1988). Prosencephalic asymmetries in  Lemuridae. Brain, Behavior and Evolution. Brain Behav Evol.

[B56] Radespiel U, Cepok S, Zietemann V, Zimmermann E (1998). Sex-specific usage patterns of sleeping sites in grey mouse lemurs (*Microcebus murinus*) in Northwestern Madagascar. Am J Primatol.

[B57] Radespiel U, Sarikaya Z, Zimmermann E, Bruford MW (2001). Sociogenetic structure in a free-living nocturnal primate population: sex-specific differences in the grey mouse lemur (*Microcebus murinus*). Behav Ecol Sociobiol.

[B58] Niaussat MM, Petter JJ (1980). Study of the auditory sensitivity of a Malagasy prosimian *Microcebus murinus *(J.-F. Miller, 1777). Mammalia.

[B59] Zimmermann E, Altermann L, Doyle GA, Izard MK (1995). Acoustic communication in nocturnal prosimians. Creatures of the Dark: the Nocturnal Prosimians.

[B60] Zimmermann E, Lerch C (1993). The complex acoustic design of advertisement call in male mouse lemurs (*Microcebus murinus*, Prosimii, Primates) and sources of its variation. Ethology.

[B61] Hafen T, Neveu H, Rumpler Y, Wilden I, Zimmermann E (1998). Acoustically dimorphic advertisment calls separate morphologically and genetically homogenous populations of the grey mouse lemur (*Microcebus murinus*). Folia Primatol.

[B62] Zimmermann E, Vorobieva E, Wrogemann D, Hafen T (2000). Use of vocal fingerprinting for specific discrimination of gray (*Mirocebus murinus*) and rufous mouse lemurs (*Microcebus rufus*). Int J Primatol.

[B63] Braune P, Schmidt S, Zimmermann E (2005). Spacing and group coordination in a nocturnal primate, the golden brown mouse lemur (*Microcebus ravelobensis*): the role of olfactory and acoustic signals. Behav Ecol Sociobiol.

[B64] Scheumann M, Deichsel G, Zimmermann E (2007). Context-specific calls signal infants' needs in a strepsirrhine primate, the gray mouse lemur (*Microcebus murinus*). Dev Psychobiol.

[B65] Ghirlanda S, Vallortigara G (2004). The evolution of brain lateralization: a game-theoretical analysis of population structure. Proc Roy Soc B-Biol Sci.

[B66] Spinozzi G, Castorina MG, Truppa V (1998). Hand preferences in unimanual and coordinated-bimanual tasks by tufted capuchin monkeys (*Cebus apella*). J Comp Psychol.

[B67] Wagner TD, Luan Phan K, Liberzon I, Tylor SF (2003). Valence, gender, and lateralization of functional brain anatomy in emotion: a meta-analysis of findings from neuroimaging. Neuroimage.

[B68] Schoenwiesner M, Rübsamen R, Von Cramon DY (2005). Hemispheric asymmetry for spectral and temporal processing in the human antero-lateral auditory belt cortex. Eur J Neurosci.

[B69] Ghazanfar AA, Smith-Rohrberg D, Hauser MD (2001). The role of temporal cues in rhesus monkey vocal recognition: Orienting asymmetries to reversed calls. Brain Behav Evolut.

[B70] Altenmüller E, Schürmann K, Lim VK, Parlitz D (2002). Hits to the left, flops to the right: different emotions during listening to music are reflected in cortical lateralisation patterns. Neuropsychologia.

[B71] Shaywitz BA, Shaywitz SE, Pugh KR, Constable RT, Skudlarski P, Fulbright RK, Bronen RA, Fletcher JM, Shankweiler DP, Katz L (1995). Sex differences in the functional organization of the brain for language. Nature.

[B72] Kansaku K, Yamaura A, Kitazawa S (2000). Sex differences in lateralization revealed in the posterior language areas. Cereb Cortex.

[B73] Davatzikos C, Resnick SM (1998). Sex differences in anatomic measures of interhemispheric connectivity: Correlations with cognition in women but not men. Cereb Cortex.

[B74] De Lacoste M-C, Woodward DJ (1988). The corpus callosum in nonhuman primates. Determinants of size. Brain Behav Evol.

[B75] Holloway RL, Heilbroner P (1992). Corpus callosum in sexually dimorphic and nondimorphic primates. Am J Phys Anthropol.

[B76] Aydinlioğlu A, Arslan K, Cetin Rağbetli M, Riza Erdoğan AR, Keles P, Diyarbakirli S (2000). Sex differences in dog corpus callosum. Eur J Morphol.

[B77] Cowell PE, Denenberg VH, Rogers LJ, Andrew RJ (2002). Development of laterality and the role of the corpus callosum in rodents and humans. Comparative Vertebrate Lateralization.

[B78] Wrogemann D, Radespiel U, Zimmermann E (2001). Comparison of reproductive characteristics and changes in body weight between captive populations of rufous and gray mouse lemurs. Int J Primatol.

[B79] Braune P (2007). Acoustic variability and its biological significance in nocturnal lemurs.

[B80] Bortz J, Lienert GA, Boehnke K (2000). Verteilungsfreie Methoden in der Biostatistik.

